# Is Innovation Worth the Price? Cost-Effectiveness Analysis in Interventional Oncology

**DOI:** 10.1007/s00270-025-04213-5

**Published:** 2025-09-28

**Authors:** Ludovico Cavallaro, Carla Rognoni

**Affiliations:** Center for Research on Health and Social Care Management (CERGAS), SDA Bocconi School of Management, Via Sarfatti, 10, 20136 Milan (MI), Italy

**Keywords:** Health technology assessment (HTA), Full economic evaluations, Cost-effectiveness analysis (CEA), Interventional oncology (IO), Transarterial radioembolization (TARE), Sorafenib

## Abstract

Cost-Effectiveness Analysis (CEA) plays a crucial role in Health Technology Assessment (HTA) by informing healthcare decision-making on resource allocation. A rigorous CEA requires selecting appropriate comparators, assessing costs comprehensively, evaluating clinically relevant outcomes, and employing robust modeling techniques to determine an intervention’s value for money. This is particularly relevant in interventional oncology, where innovative yet costly procedures must demonstrate long-term benefits to justify adoption. Economic evidence is essential to guide healthcare decision-making by balancing clinical effectiveness with the efficient use of resources. However, CEA findings must be interpreted within the broader HTA framework, incorporating ethical, organizational, and social considerations. Using the analysis by Rognoni et al. as a case study comparing transarterial radioembolization (TARE) and sorafenib for the treatment of liver tumors, this paper illustrates how CEA supports evidence-based healthcare policies.

## Introduction

The scarcity of healthcare resources and the challenge of allocating them efficiently are pressing concerns in most industrialized countries. This issue has gained increasing prominence in recent years, driven by a combination of demographic, technological, and social factors [1]. Advances in medical science and technology have significantly improved life expectancy, leading to an aging population and a rising burden of multimorbidity that demands more complex and sustained care. At the same time, declining fertility rates and a rising old-age dependency ratio exert further pressure on already constrained resources. In parallel, the rapid pace of innovation has expanded the range of diagnostic and therapeutic interventions, enabling treatment of a broader spectrum of health conditions. Moreover, rising health literacy and wider access to medical information have heightened public awareness and expectations. Patients are now more informed about treatment options and increasingly demand timely, high-quality care.

In publicly funded healthcare systems, resource allocation is typically governed by the principle of solidarity, ensuring that access to care is based on medical need rather than financial means [2]. In this context, ‘need’ is defined as a patient’s capacity to benefit from an intervention. This definition underscores the importance of evaluating healthcare programs based on their effectiveness in improving health outcomes [3]. However, as healthcare demand continues to rise, resource constraints become increasingly pressing. This situation calls for the adoption of decision-making frameworks that not only consider clinical effectiveness but also assess whether interventions provide good value for money by maximizing health benefits relative to costs.

The debate on the use of scientific evidence in decision-making has evolved significantly over time, leading to the widespread adoption of evidence-based practices across many public sectors [4]. In healthcare, the principles of evidence-based medicine, initially applied to clinical practice, have expanded into management and policy-making, increasing empirical research aimed at supporting decision-making [5]. One of the most established frameworks integrating scientific evidence into healthcare policy is Health Technology Assessment (HTA) [6].

HTA is defined as “a multidisciplinary process that uses explicit methods to determine the value of a health technology at different points in its lifecycle. The purpose is to inform decision-making in order to promote an equitable, efficient, and high-quality health system” [7]. By synthesizing evidence from various disciplines, HTA informs resource allocation decisions, such as the inclusion of pharmaceuticals in formularies, the determination of insurance coverage, and the development of clinical guidelines [8]. While initially focused on pharmaceuticals, HTA now encompasses medical devices, surgical procedures, diagnostic programs, public health interventions, and disease management strategies [9].

HTA is a structured and multidimensional process of analysis and decision-making. It is structured because it requires the systematic collection and analysis of data to support the evaluation of healthcare technologies. It is multidimensional because the impact of a technology must be assessed across multiple domains [7]. Healthcare policy decisions have significant implications, affecting both individual well-being and the overall sustainability and equity of the healthcare system. For this reason, HTA considers not only clinical and economic impact, which together form the basis of full economic evaluations (FEEs), but also organizational, ethical and social aspects [10].

Among FEEs, cost-effectiveness analysis (CEA) is extensively used to assess the costs and health outcomes of alternative interventions. Its application extends across various medical domains, including interventional oncology, where resource-intensive procedures necessitate rigorous evaluation to substantiate evidence-informed decision-making.

Following an outline of the role of FEEs in healthcare decision-making and their methodological foundations, this article focuses on the application of CEA in interventional oncology through a case study [11] comparing transarterial radioembolization (TARE) and sorafenib for the treatment of hepatocellular carcinoma (HCC).

## Full Economic Evaluations

FEEs provide a structured framework for comparing alternative courses of action in terms of both their costs and consequences [12]. While they do not replace clinical evaluations, they complement them by incorporating cost considerations into the decision-making process. Clinical studies primarily establish the efficacy and safety of interventions; economic evaluations build on this foundation to assess whether the health benefits justify the associated costs.

There are three main types of FEEs [12, 13]: cost-effectiveness analysis (CEA), cost-utility analysis (CUA), and cost-benefit analysis (CBA). While all three measure costs in monetary terms, they differ in how they assess health outcomes:

- CEA compares interventions based on physical health units, such as life years gained. This method is widely used because it aligns with clinical measures familiar to healthcare professionals. However, a major limitation is that it only allows comparisons between interventions with the same type of health outcome.
- CUA addresses this limitation by measuring outcomes in quality-adjusted life years (QALYs), which incorporate both life expectancy and quality of life. This allows for comparisons across different interventions and conditions by providing a standardized measure of health benefit.
- CBA takes a different approach by expressing both costs and health benefits in monetary terms. This allows direct comparisons between healthcare and non-healthcare investments. However, assigning a financial value to health outcomes raises ethical and methodological concerns, which limits its widespread use.

It is worth mentioning that, in practice, the distinction between CEA and CUA is often blurred. The term ‘cost-effectiveness analysis’ is frequently used in the literature as a broad category encompassing both approaches, leading to inconsistencies in definitions and interpretations [14]. As a result, studies that incorporate QALYs are often labeled as CEAs, even though they technically are CUAs. To avoid confusion, in this article, and particularly in the case study that follows, we explicitly consider CEA as an analysis that measures health outcomes in physical health units.

## Cost-Effectiveness Analysis in Interventional Oncology: The Case Study of Transarterial Radioembolization (TARE) and Sorafenib for the Treatment of Hepatocellular Carcinoma (HCC)

Conducting and interpreting CEAs requires careful consideration of the complexities underlying their methods and results. Key decisions, such as which cost items to include, how to measure them, and how to assess clinical outcomes, significantly impact conclusions. Cost-effectiveness can vary depending on whether the analysis is conducted from the perspective of a national healthcare system, a private insurer, or a broader societal perspective that accounts for productivity losses and patients’ out-of-pocket expenses. Additionally, the choice of economic model, from simple decision trees to more complex Markov multi-state models, influences the robustness and generalizability of findings. Given the breadth of these methodological nuances, this discussion offers a conceptual overview rather than an exhaustive analysis. For a more comprehensive exploration of CEA methodologies, readers may consult a foundational reference in economic evaluation [12].

This section presents a case study [11] applying CEA to interventional oncology, comparing TARE and sorafenib for the treatment of HCC. By examining the study’s analytical structure, cost and effectiveness assessments, and interpretation of findings, the case study illustrates the practical steps involved in conducting a CEA within a clinical setting where innovative treatments continue to emerge and healthcare resources remain constrained.

### Identifying and Comparing Alternatives

The first and most critical step in any CEA is selecting the appropriate interventions for comparison, as this choice profoundly influences how results are interpreted. A new treatment is typically compared against the best available standard of care or a commonly used therapy in clinical practice, possibly the least costly option. In cases where no established alternative exists, the comparator may be a “no treatment” scenario, recognizing that even the absence of intervention carries associated costs [12].

This analysis focused on the comparison between TARE and sorafenib, an oral antineoplastic agent. At the time of the study, TARE had emerged as a locoregional treatment option for patients with unresectable HCC, while sorafenib was the established systemic therapy in this setting [11, 15]. To ensure comparability between patient groups eligible for both TARE and sorafenib, the analyzed population included intermediate-stage patients who had failed transarterial chemoembolization (TACE), as well as advanced-stage patients according to the Barcelona Clinic Liver Cancer staging system with portal vein thrombosis (PVT), no extrahepatic spread, and preserved liver function. Given these overlapping indications, TARE was considered a potential alternative to sorafenib, raising the question of whether it could offer comparable or superior outcomes in a cost-effective manner. Although TARE showed promising clinical efficacy with a good safety profile in phase II studies and registries [16], it had not yet been formally integrated into clinical guidelines. Its use was limited to specialized centers and required a multidisciplinary team, making it important to assess whether the additional complexity and costs were justified by better health outcomes. A CEA was therefore necessary to determine whether TARE provided good value for money compared to sorafenib.

### Healthcare Resource Consumption and Costs

Following the selection of interventions, the next step is cost assessment, which depends on the analytical perspective, data availability, intervention complexity, and the resources available to the research team. Two primary methods are commonly employed:

- Micro-costing, or the bottom-up approach, involves identifying each resource used in delivering an intervention and assigning a monetary value to it. This method provides highly detailed data, enabling precise identification of resource utilization and cost variation among patients. However, it is time-intensive and relies on access to granular data from hospital information systems or direct observation.
- Gross costing, or the top-down approach, aggregates total expenditures over a defined period and distributes costs across different procedures or services. While this method simplifies data collection, it may obscure variability in resource consumption among procedures and patients, leading to less precise cost estimates.

In the case study, cost analysis was conducted from the perspective of the Lombardy Regional Healthcare Service in Northern Italy, focusing on direct medical costs. These included hospital admissions, laboratory tests, imaging, and pharmaceuticals. Cost data for hospitalizations and outpatient healthcare services were obtained from diagnosis-related groups (DRGs) reimbursement rates and official tariffs for Lombardy, respectively. Given the regional healthcare service's perspective, the analysis excluded direct non-healthcare costs (e.g., out-of-pocket expenses) and indirect costs (e.g., productivity losses).

The treatment pathway for TARE considered a first oncology visit, a procedure simulation (DRG 203), laboratory tests, and the treatment itself (DRG 409). After one month, a follow-up oncology visit and laboratory tests are repeated, including an abdomen computerized tomography (CT) scan. During follow-up, an oncology visit, laboratory tests, and an abdominal CT scan are performed every three months.

The treatment pathway for sorafenib considered, following an initial oncology visit and laboratory tests, the monthly administration of sorafenib (112 tablets of 200 mg each; hospital cost: 3,536.17€) to each patient until disease progression. At the time of the analysis in Italy, a payment-by-result scheme was in place for sorafenib, whereby the manufacturer was expected to reimburse the cost of up to two packages in case of non-response within the first two months. To determine treatment response and eligibility for reimbursement, an abdominal CT scan is therefore performed two months after initiating sorafenib. Otherwise, follow-up is the same as per TARE treatment.

Subsequent treatments were identified from the cohort data (derived from the retrospective case–control study presented in the following section) and incorporated into the model. Liver decompensation was reported by the clinical experts as the most economically and clinically relevant adverse event, leading to hospitalization (DRG 464). Liver transplantation costs are based on the DRG tariff (DRG 408), while lifetime costs after the intervention were derived from the literature [17] and are estimated at 6,182€ per year. The detailed breakdown of healthcare resources and associated costs used in the model is presented in Table 1.

**Table 1:** Healthcare resources and costs used in the model. Source: Adapted from [11]

Exam/procedure/DRG	Timing	Code	Cost (€)	Reference
CT examination (abdomen)	every 3 months	88.01.5	137.23	Regional Healthcare Service price list
First visit	1 time	89.7B.6	22.50	Regional Healthcare Service price list
Control visit	every 3 months	89.01.F	17.90	Regional Healthcare Service price list
Blood count	every 3 months	90.62.2	4.05	Regional Healthcare Service price list
Creatinine	every 3 months	90.16.3	1.70	Regional Healthcare Service price list
Sodium	every 3 months	90.40.4	1.70	Regional Healthcare Service price list
Potassium	every 3 months	90.37.4	1.70	Regional Healthcare Service price list
Calcium	every 3 months	90.11.4	1.70	Regional Healthcare Service price list
Prothrombin time	every 3 months	90.75.4	2.60	Regional Healthcare Service price list
Albumin	every 3 months	90.05.1	2.90	Regional Healthcare Service price list
Bilirubin	every 3 months	90.10.4	1.55	Regional Healthcare Service price list
Alpha-Fetoprotein	every 3 months	90.05.5	11.05	Regional Healthcare Service price list
Alanine amino transferase (alt) (gpt)	every 3 months	90.04.5	1.70	Regional Healthcare Service price list
Gamma-glutamyl transpeptidase	every 3 months	90.25.5	1.70	Regional Healthcare Service price list
Alkaline phosphatase	every 3 months	90.23.5	1.70	Regional Healthcare Service price list
Sorafenib	7.5 and 8.1 months duration for intermediate and advanced stages, respectively Following TARE: 30.3% of patients in intermediate stage, 20% of patients in advanced stage		3,787	Monthly Hospital cost
TARE simulation	1 procedure per patient	203	4,052	Regional DRG reimbursement
TARE	1.1 procedures per patient in intermediate stage, 1.02 procedures per patient in advanced stage	409	9,510	Regional DRG reimbursement
TACE	Following TARE: 18.3% of patients in intermediate stage, 2.2% of patients in advanced stage; Sorafenib 6.6% of patients in intermediate stage, 2.6% of patients in advanced stage	203	4,052	Regional DRG reimbursement
RFA/PEI or liver resection	Following TARE: 5.5% in intermediate stage; Sorafenib: 10.5% of patients in intermediate stage, 1.3% of patients in advanced stage	192	7,549	Regional DRG reimbursement
Radiotherapy	TARE: 2.2% in advanced stage	409	4,041	Regional DRG reimbursement
Hospitalization for liver decompensation	TARE: 19.4% of patients in intermediate stage, 43% of patients in advanced stage; Sorafenib: in intermediate stage 17.4%, in advanced stage 31%	464	1,688	Regional DRG reimbursement
Liver transplantation	Following TARE: 3.7% of patients in intermediate stage	480	68,027	Regional DRG reimbursement
Liver transplantation (yearly cost after intervention)	Following TARE: 3.7% of patients in intermediate stage		6,182	[17]

### Effectiveness Assessment and Markov Modeling

The primary effectiveness indicator used to evaluate health technologies and programs is survival, typically measured in life-years gained. However, when direct measurement of survival is not feasible, alternative outcome measures may be necessary. The general principle is to adopt intermediate outcomes when there is a well-established relationship with survival or another relevant final outcome, or when the intermediate outcome has intrinsic clinical value [18].

The primary source of effectiveness data in economic evaluations comes from the medical literature: experimental studies (such as randomized clinical trials, RCTs) and real-world studies (such as observational studies). When clinical data are unavailable, expert opinion may be considered, though it is less reliable [12].

In the case study, a retrospective case–control design was employed to analyze patient-level data from three oncology centers in Italy. Patients receiving TARE were matched one-to-one with those treated with sorafenib based on three clinically relevant parameters: presence of PVT, performance status, and number of tumor nodules. This matching process resulted in two comparable cohorts of 154 patients each.

A Markov multi-state model was then employed to estimate long-term costs and health outcomes [19]. This modeling approach, widely used in oncology, allows patients to transition between different health states such as stable disease, disease progression, death due to HCC, or death from other causes at predefined time intervals, typically one or three months. For intermediate-stage HCC, an additional health state accounted for the possibility of liver transplantation. Transition probabilities were derived from the matched retrospective cohort, ensuring that the model accurately reflected observed survival outcomes, including overall survival (OS) and progression-free survival (PFS).

The model simulated a hypothetical cohort of patients starting treatment with either TARE or sorafenib and followed them over time as they moved through the defined health states. At each cycle, patients accumulated costs and life years based on the time spent in each state and the associated resource use. This structure enabled the estimation of total costs and effectiveness over a lifetime horizon, incorporating not only survival outcomes but also treatment sequences, follow-up care, and major events such as hospitalization or transplantation. In this way, the analysis provided comprehensive estimates of the long-term clinical and economic impact of the two treatment strategies.

### Interpreting Results and Decision Rules

Once the costs and effectiveness of the alternatives have been estimated, the next step is to determine whether one intervention offers better value for money than the other.

Consider two programs, A and B, where A represents an innovative intervention and B the existing standard of care. Let C_A_ and C_B_ denote the costs of programs A and B, respectively, while E_A_ and E_B_ represent their effectiveness. The comparison between these programs may lead to three possible scenarios:

- Dominance of Program A: If A is less costly and more effective than B (C_A_ < C_B_; E_A_ > E_B_), program B is dominated and should be abandoned. In this case, program A is also considered cost-saving, as it improves health outcomes while freeing up resources that can be allocated elsewhere.
- Dominance of Program B: If A is more costly and less effective than B (C_A_ > C_B_; E_A_ < E_B_), program A is dominated and should be discarded.
- Incremental Cost-Effectiveness Ratio (ICER): If A is more costly but also more effective than B (C_A_ > C_B_; E_A_ > E_B_), further evaluation is required. This is done by calculating the ICER, which quantifies the additional cost per unit of health benefit gained.

The ICER is obtained by dividing the difference in costs by the difference in effectiveness. Once the ICER is calculated, the key question is whether the intervention is eligible for funding. The answer depends on whether the programs being compared are mutually exclusive or non-mutually exclusive.

Mutually exclusive programs refer to interventions where only one option can be implemented, replacing the existing alternative. In this case, the ICER is compared against an acceptability threshold to determine funding eligibility. Programs with an ICER below this threshold are considered for adoption. Many national health systems use cost-effectiveness thresholds to determine how much they are willing to pay for each additional life year gained or QALY. Some countries, such as the United Kingdom, have official bodies like the National Institute for Health and Care Excellence (NICE) that establish or recommend such thresholds [20]. Others, including Italy, do not have a universally accepted threshold. However, estimates in the literature [21, 22] suggest a range between €20,000 and €60,000.

On the other hand, non-mutually exclusive programs are independent interventions that can be implemented within available financial resources. These programs are ranked based on their average cost-effectiveness ratio (total cost divided by total effectiveness), prioritizing those with the lowest ratio. The final selection is dictated by budget constraints, funding as many programs as possible without exceeding financial limits.

Turning to the findings of the case study, for patients in the intermediate stage of HCC, the analysis estimated a mean survival of 2.531 years with TARE, compared to 1.575 years with sorafenib. The mean costs per patient were €31,071 in the TARE group and €29,289 in the sorafenib group, resulting in an ICER of €1,865 per life-year gained. Since this ICER is well below the commonly cited thresholds for Italy, TARE was identified as a cost-effective strategy for patients in the intermediate stage of HCC.

For advanced-stage patients, the model estimated a mean survival of 1.445 years with TARE and 1.306 years with sorafenib. The estimated mean costs were €21,961 per patient for TARE and €30,750 per patient for sorafenib. In this case, TARE dominated sorafenib, as it was both less expensive and more effective. A summary of the model results is provided in Table 2.

**Table 2:** Summary of the model results. Source: Adapted from [11]

Disease stage	Strategy	Cost (€)	Δ Cost (€)	LYs	ΔLYs	ICER (€/LY)
Intermediate	TARE	31,071	1,782	2.531	0.956	1,865
	Sorafenib	29,289		1.575		
Advanced	TARE	21,961		1.445	0.139	Dominant
	Sorafenib	30,750	8,788	1.306		

To assess the robustness of these findings, sensitivity analyses were conducted by varying key parameters, such as cost estimates, transition probabilities, and the time horizon of the model, within plausible ranges. The results indicated that only a significant shift in certain parameters, such as the number of TARE procedures per patient or extending the time horizon, could raise the ICER above €50,000 per life-year gained. However, as each patient is unlikely to undergo more than 1.5 TARE treatments on average, the findings appear robust within the practical context of HCC management.

## Conclusions

This paper highlights the role of FEEs, particularly CEA, as a fundamental tool within HTA to inform healthcare decision-making. A well-structured CEA requires the careful selection of comparators, a comprehensive assessment of costs, the identification of clinically relevant outcomes, and the use of robust modeling techniques to determine whether an intervention provides good value for money and justifies its adoption within a healthcare system.

CEA is particularly relevant in interventional oncology, where innovative procedures often involve high upfront costs but may provide long-term benefits in survival and disease management. As oncological treatments become increasingly complex, economic evidence plays a crucial role in optimizing both clinical outcomes and resource allocation. However, CEA findings must be interpreted within the broader HTA framework, which also considers ethical, organizational, and social implications that influence real-world decision-making.

The case study demonstrates how economic evaluations can guide resource allocation by identifying treatments that deliver the best value for money. While specific results may vary across healthcare settings, the methodological principles of CEA remain broadly applicable, reinforcing its importance in shaping evidence-based healthcare policies.
